# Effect of high-flux, low-energy He^+^ ion irradiation on Ta as a plasma-facing material

**DOI:** 10.1038/srep39746

**Published:** 2016-12-23

**Authors:** T. J. Novakowski, J. K. Tripathi, A. Hassanein

**Affiliations:** 1 Center for Materials Under eXtreme Environment (CMUXE), School of Nuclear Engineering, Purdue University, West Lafayette, IN 47907, USA

## Abstract

The goal of this work is to assess Ta as a potential plasma-facing material for future fusion reactors in terms of its response to high-flux, low-energy He^+^ ion irradiation. Ta samples were irradiated with 100 eV He^+^ ions at various fluences up to 3.5 × 10^25^ ions m^−2^ while simultaneously heated at constant temperatures in the range 823–1223 K. SEM studies show that irradiated Ta surfaces undergo significant morphology changes that have a strong dependence on both ion fluence and sample temperature. Optical reflectivity complements SEM and demonstrates a vertical growth of surface structures with increasing fluence. *Ex situ* XPS and XRD both show significant oxidation of the irradiated Ta surfaces, giving further qualitative information on the extent of surface modification. Overall, these irradiation-induced structures on Ta are similar to early-stage “fuzz” structures observed in W. However, Ta exhibits a higher fluence threshold for structure formation. While Ta may have less desirable bulk properties (e.g., thermal conductivity) when compared to W, its higher resilience to He^+^ ion-induced surface modification suggests that surface thermal and mechanical properties may not degrade as quickly in extreme fusion environments; this quality may be a redeeming factor for Ta as a plasma-facing material.

Over the past several decades, a variety of materials have been considered for use as plasma-facing components (PFCs) in nuclear fusion reactors. Namely for tokamak reactor designs, such as the international thermonuclear experimental reactor (ITER), PFCs will be subjected to high fluxes of helium (He), hydrogen (H) isotopes, neutrons, and immense steady state and transient heat fluxes^1^. Accordingly, PFC materials need to have relatively high thermal conductivities and low sputtering/erosion rates. Currently, tungsten (W) is considered the leading candidate material for PFCs and has been the most widely studied material in the fusion community for the past several years. Its high melting point and thermal conductivity ensure that the material can withstand and efficiently transfer large heat loads, and its relatively low surface sputtering rates minimize plasma contamination as a result of surface erosion from the fusion plasma. However, for high fluxes and fluences of low-energy He^+^ ion irradiation, in combination with elevated sample temperatures, W surfaces are known to develop significant surface nanostructuring including surface pores^2^, bubbles^3^, and nano-scale tendril-like structures commonly referred to as “fuzz”^4^,^5^. This “fuzz” is essentially a tangled or conglomerated mass of fine surface tendrils that is relatively fragile (easily removed by abrasion) and exhibits a very high porosity.

As a product of the deuterium-tritium (D-T) fusion reaction, He only constitutes a relatively small portion of the fusion plasma. However, He plays a more active role in surface structure development of irradiated metals than H or D^6^,^7^, largely due to its stronger trapping behavior at radiation-induced defects^8^. Interestingly, these W nanostructures are known to occur even when the He^+^ ions are well below the sputtering and displacement thresholds of W, suggesting that structure formation is somehow driven by the accumulation and growth of over-pressurized sub-surface He bubbles^9^. There is still some debate on the actual driving mechanisms involved, but theories include surface roughening from mobile He clusters^10^, enhanced W self-interstitial migration along immobile He clusters^11^, and even viscoelastic bubble flow^12^. While still not completely understood, there are serious concerns that these fuzzy nanostructures could have a negative impact on fusion reactor performance. It has been shown that these fuzzy structures demonstrate significantly reduced thermal conductivity^13^, have increased H isotope retention (effectively lowering fuel economy and safety)^14^, and may be easily removed through abrasive action or transient plasma events^15^. If these fuzzy structures were to be removed from the W surfaces, they would act as high-Z impurities in the plasma, leading to plasma quenching as a result of radiative energy loss^16^.

In light of these potentially detrimental surface structures observed in W, recent studies^17^ have shown that alternative refractory metals could potentially be used as PFCs due to their similar thermal, mechanical, and activation properties. However, there are only a few preliminary studies investigating the response of these alternative refractory metals to high fluxes of He and H isotopes. It has been shown that under these similar He^+^ ion irradiation conditions, significant nanostructuring is also observed in most other refractory metals such as molybdenum (Mo)^18^,^19^, niobium (Nb)^20^, and vanadium (V)^21^. However, similar preliminary temperature-dependent studies in Ta^22^ show that Ta does not form the same high aspect ratio surface structures as observed in W, Mo, Nb, and V; rather, Ta only forms shallow surface pores when irradiated with high-flux, low-energy He^+^ ion irradiation. Nevertheless, it was hypothesized that higher aspect ratio surface structures (similar to fuzz) could be formed at higher fluences since surface pores are known to be a precursor to fuzz formation in W^9^. To our knowledge, fuzz formation has never been observed in Ta except for in a very recent study^23^ that showed for the first time fuzz formation in Ta using sufficiently high He^+^ ion fluences.

The primary motivation for this systematic study is to assess the relevance of Ta as a PFC material in future fusion reactors in terms of its response to high-flux, low-energy He^+^ ion irradiation. Even if Ta is found to form surface structures at relatively high He^+^ ion fluences, preliminary studies^22^,^23^ suggest that Ta would have a higher fluence tolerance for surface structure formation. While Ta has an initially lower thermal conductivity than W, and is therefore deemed less ideal as a PFC material, a higher fluence threshold for structure formation would suggest that Ta surface’s thermal conductivity is less likely to degrade over the lifetime of reactor operation. If demonstrated, this improved resilience to surface structure formation could be a redeeming quality for Ta as a PFC. Even if the bulk material properties of Ta are not as desirable as W, Ta may be applied in the form of a coating to take advantage of W’s bulk properties and Ta’s surface properties. As a secondary motivation, studying the effects of high-flux, low-energy He^+^ ion irradiation on surfaces of non-W materials could give some insight into the physical mechanisms of fuzz and nanostructure formation. Therefore, even if Ta is still determined to be an undesirable material for PFCs, the studies presented in this work will be advantageous for the elucidation of He^+^ ion-induced structure formation processes.

## Results and Discussion

### Atomic force microscopy (AFM), electron backscatter diffraction (EBSD), and scanning electron microscopy (SEM)

**Figure 1:**
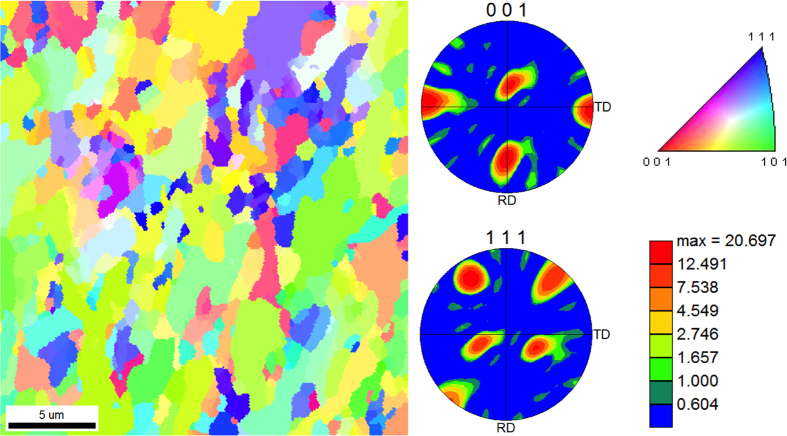
Left: typical EBSD grain orientation map of a virgin, polished Ta sample. Right: <001> and <111> pole figures of the same region.

**Figure 2:**
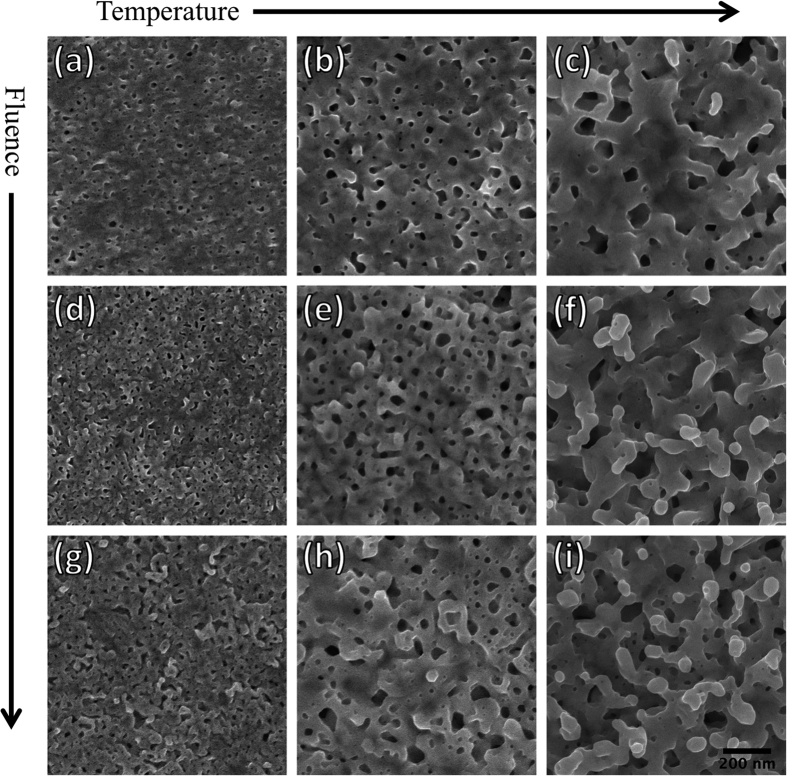
SEM micrographs of Ta samples irradiated with 100 eV He^+^ ions at various fluences and temperatures: (**a–c**) 4.3 × 10^24^ ions m^−2^ at 823, 1023, and 1223 K, respectively; (**d–f**) 8.6 × 10^24^ ions m^−2^ at 823, 1023, and 1223 K, respectively; (**g–i**) 1.7 × 10^25^ ions m^−2^ at 823, 1023, and 1223 K, respectively.

Preliminary surface characterizations of the virgin, mirror-polished samples were performed using atomic force microscopy (AFM) and electron backscatter diffraction (EBSD) to determine the material’s initial surface roughness and average grain size, respectively. AFM revealed that the average surface roughness for polished samples is ~2 nm. Calculated average grain size, using the EBSD grain orientation map in Fig. 1, is ~3 μm. Note that most of the observed grains are irregularly shaped and exhibit some grain deformation. Such grain deformation can be attributed to the cold rolling process^24^ used during the material’s preparation, making it difficult to perform accurate stereology and obtain actual grain distributions. Nevertheless, this study gave us an idea of typical Ta grain size to decouple grain boundary effects from post-irradiation surface morphologies. This is useful to determine whether the structure growth characteristics are solely element-dependent or if other material parameters (like grain size or grain boundary density) could also play a role. Grain boundaries act as sinks for interstitials and vacancies^25^ and may help to improve the irradiation-tolerance of materials. In fact, recent studies^26^ have shown that for W under high-flux He^+^ ion irradiation, nano-grained W demonstrates a decrease in He bubble number density due to the increased density of grain boundaries in the material. Additionally, Fig. 1 shows both < 001 > and < 111 > pole figures (PFs) to display crystallographic textures. The PFs show no significant texturing or preferential orientation. Particularly for body-centered cubic (BCC) metals such as Ta, it is well known that crystallographic texture may be difficult to control during the cold rolling process^24^. Figure 2 shows a collection of high-resolution SEM micrographs from Ta samples irradiated with 100 eV He^+^ at various fluences and temperatures, effectively creating a parameter “map” of Ta surface evolution under low-energy He^+^ irradiation. The first three micrographs (Fig. 2(a–c)) show Ta surfaces irradiated to a fluence of 4.3 × 10^24^ ions m^−2^ at temperatures of 823, 1023, and 1223 K, respectively. It should be noted that the irradiation conditions were identical to samples presented in our earlier work on Ta^22^ and the resulting structures are expectedly similar. For all these lower-fluence samples, the main mode of surface damage is pores/pinholes that increase and diameter (and decrease in number density) with increasing temperature. This effect is attributed to the enhanced mobility of sub-surface He clusters at higher temperatures, effectively causing He clusters to agglomerate into larger clusters. These larger clusters, in turn, create larger pinholes when they escape from the Ta surface. It should be noted that for the 1223 K case, a few nano-scale surface protrusions were also observed (Fig. 2(c)). Moving on to a fluence of 7.6 × 10^24^ ions m^−2^ (Fig. 2(d–f)), the sizes of surface pores at each temperature are reasonably consistent with their lower-fluence counterparts. At least for the samples irradiated at 823 and 1023 K, Fig. 2(d,e) respectively, the number density of pores is noticeably larger. Although it cannot be quantitatively determined with SEM, the surface roughness at each temperature appears to increase with ion fluence; this will later be confirmed with optical reflectivity. Most notably, the sample irradiated at 1223 K shows the early stages of nano-tendril formation, suggesting that fuzz will likely form at this temperature at higher fluences. At the highest fluence of 1.7 × 10^25^ ions m^−2^ (Fig. 2(g–i)), surface structures are yet again more pronounced and suggest a larger surface roughness. At the highest temperature of 1223 K (Fig. 2(i)), nano-tendrils are now apparent and appear to be vertically protruding from the surface. At the lower temperatures of 823 and 1023 K (Fig. 2(g,h), respectively), we find what may be the very early stages of tendril growth. This suggests that fuzz may be able to be formed at these temperatures; however, additional experiments at substantially higher fluences would be needed to confirm this.

**Figure 3:**
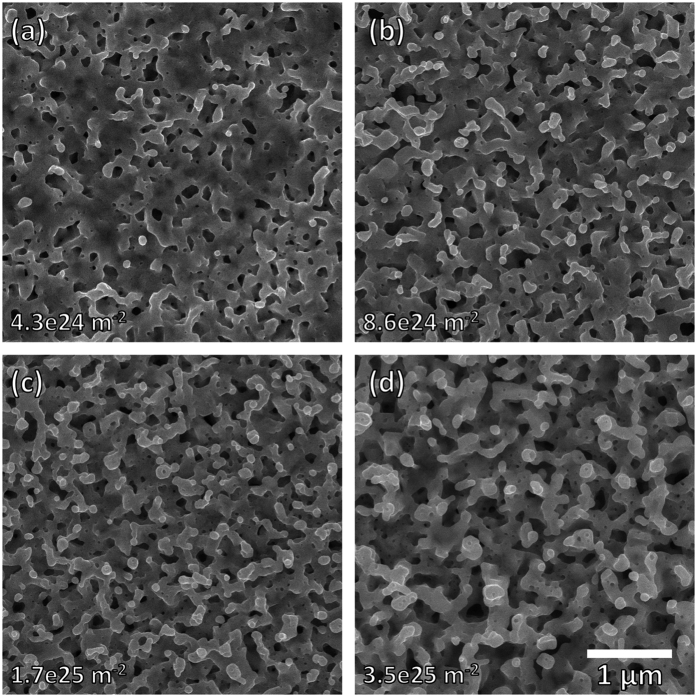
SEM micrographs surfaces irradiated at 1223 K with 100 eV He^+^ ions to fluences of (**a**) 4.3 × 10^24^, (**b**) 8.6 × 10^24^, (**c**) 1.7 × 10^25^, and (**d**) 3.5 × 10^25^ ions m^−2^.

**Figure 4:**
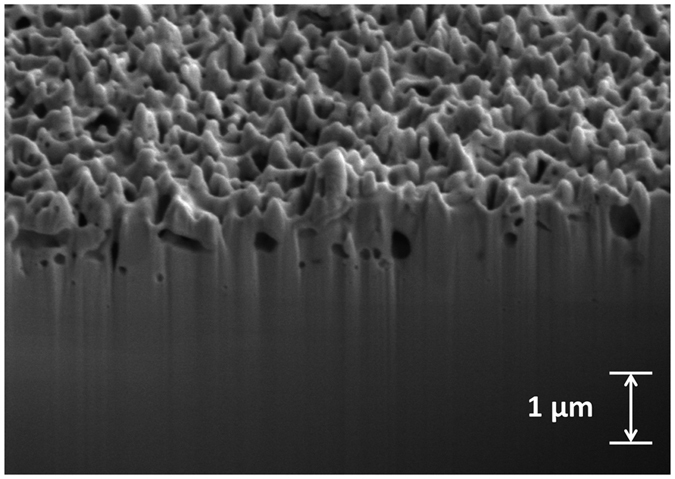
Cross-sectional SEM micrograph of a nanostructured Ta sample irradiated with 100 eV He^+^ ions at a sample temperature of 1223 K and to a fluence of 3.5 × 10^25^ ions m^−2^.

Because of the fuzz-like structures observed in Fig. 2(c,f,i), we performed one additional irradiation at 1223 K to a total fluence of 3.5 × 10^25^ ions m^−2^. Figure 3 shows the surface evolution for these samples irradiated at 1223 K. Between the sample irradiated at 4.3 × 10^24^ ions m^−2^ (Fig. 3(a)) and the sample irradiated at 3.5 × 10^25^ ions m^−2^ (Fig. 3(d)), the difference is quite dramatic. In Fig. 3(a), we only observe surface pores with some early-stage tendril growth; even the Ta surface in between the pores appears to be relatively smooth. In Fig. 3(d), there is no distinguishable surface plane and the nano-tendrils appear to be more developed with a typical tendril thickness of ~100 nm. This finding is consistent with recent results^23^, where fuzz formation was only found to occur in Ta at relatively higher He^+^ ion fluences (about one order of magnitude higher than the threshold for fuzz formation in W). To determine the height of these pronounced surface structures, cross sectional SEM analysis was performed on this highest-fluence Ta sample. The sample was prepared for cross-sectional SEM via focused ion beam (FIB) milling; the cross sectional image was taken with an FEI Nova 200 NanoLab DualBeam SEM/FIB. Figure 4 shows the cross-sectional SEM micrograph of the highest-fluence sample irradiated at 1223 K. From this image, the typical height of surface structures is on the order of a few hundred nm. From Fig. 4 there is also the occasional appearance of sub-surface voids or bubbles; these are likely remnants from the He-void complexes that are responsible for surface structure development. While the penetration depth of 100 eV He^+^ ions in Ta is quite small (~20 Angstroms, calculated from TRIM^27^ simulation), the high diffusivity and low solubility of He in metals^28^ allows implanted He to migrate deeper into the bulk and form bubbles at depths beyond the penetration depth. It should also be noted that smaller bubbles/voids are formed deeper in the bulk and larger bubbles/voids are formed closer to the surface. This indicates that the large surface pores observed at low fluences (and in between tendrils at higher fluences) are likely the result of agglomeration of bubbles/voids as they travel upward, before escaping the surface. While the highest-fluence sample was the only sample for which cross-sectional SEM analysis was performed, it is reasonable to assume that lower-fluence samples would have surface structures that are smaller in height. Overall, this cross-sectional image demonstrates that Ta does form the same high aspect ratio nanostructures that would be expected to form on W under similar high-flux, low-energy He^+^ ion irradiation conditions. However, these structures still resemble the early stages of fuzz formation in W. At comparable He^+^ ion fluences and irradiation temperatures, W will typically grow much finer fuzz tendrils with a fuzz layer thickness of several microns^29^. This enhanced resilience to radiation-induced surface structuring in Ta is actually more consistent with the radiation resistance of ultrafine-grained W^30^. Therefore, these irradiation experiments suggest that pure Ta has a significantly higher fluence threshold for structure formation than pure W. As mentioned previously, even though Ta initially has a lower thermal conductivity than W, its resilience to He^+^ ion-induced surface structure formation could mean that Ta thermal conductivity might degrade less quickly in fusion reactor environments, thus partially redeeming this material as a PFC. However, dedicated studies investigating the degradation of thermal conductivity in irradiated Ta surfaces would be needed to substantiate these claims.

As mentioned in the introduction, the secondary motivation for this work is to examine how differences in structure growth in Ta (and other materials), compared to W, could give some insight into the growth mechanisms of He^+^ ion-induced surface structure growth. It is interesting to note that for Ta, the temperature threshold for high aspect ratio structure formation occurs close to 1000 K. This is roughly the same temperature lower bound of the temperature window for nanostructure formation in W, which is commonly cited to be ~1000–2000 K^31^. It should also be noted that this temperature window is often reported in terms of the material’s melting temperature (i.e., ~0.3 < T/T_m_ < ~0.55, where T in the sample temperature during irradiation and T_m_ is the melting temperature) since the nanostructure formation is thought to correspond to He diffusion and related temperature-dependent mechanisms^32^. Since the melting temperature of Ta and W are very close, it seems logical that they would share a similar temperature threshold for surface structure formation. Interestingly, our previous studies with Mo^19^ have shown a temperature threshold for fuzz formation at ~873 K. When normalized for melting temperature, this threshold lies at T/T_m_ = ~0.3; this suggests that the normalized temperature window for nanostructure formation in W holds up for other materials as well. While the physical mechanisms for nanostructure growth in these materials are still not entirely known, this observed temperature window gives some valuable clues. The proposed temperature window of ~0.3 < T/T_m_ < ~0.55 is almost precisely the same temperature window where void swelling occurs in materials^33^. In fact, it is possible that void swelling could play a pivotal role in the formation of surface structures. The growth and formation of voids can act as trapping sites for interstitial sub-surface He atoms, effectively causing the formation of He-vacancy complexes that can act as nucleating points for He bubble growth. While this is likely not the sole mechanism responsible for structure growth, it is interesting to take note of this correlation.

### Optical Reflectivity

**Figure 5:**
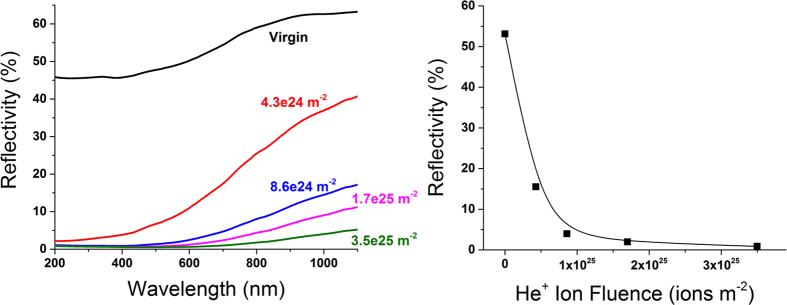
Left: specular optical reflectivity of Ta samples irradiated at 1223 K to various flucnce, referenced against a virgin, mirror-polished Ta sample for incident light wavelengths in the range 200–1100 nm. Right: optical reflectivity at 670 nm incident light wavelength as a function of ion fluence for Ta samples irradiated at 1223 K.

To quantitatively demonstrate the evolution of surface structures observed at 1223 K, optical reflectivity was performed *ex situ* on irradiated samples. Figure 5 shows the specular optical reflectivity of Ta samples irradiated at 1223 K to total fluences of 4.3 × 10^24^, 7.6 × 10^24^, 1.7 × 10^25^, and 3.5 × 10^25^ ions m^−2^, compared to the optical reflectivity of a virgin, mirror-polished Ta sample. For all irradiated samples, the optical reflectivity is significantly lower than the virgin sample for all wavelengths between 200 and 1100 nm. This suggests that irradiated surfaces have significantly increased roughness. Figure 5 also shows the optical reflectivity of 670 nm light as a function of ion fluence for all samples irradiated at 1223 K in addition to a virgin, mirror-polished sample. Since the size of surface pores and distance between tendrils does not change substantially between samples (as evidenced by Fig. 3), it is reasonable to conclude that the reduction in reflectivity is primarily due to the vertical growth of tendril structures. While optical reflectivity does not quantitatively provide depth measurements, the data presented in Fig. 5 may be used as an indirect and relative quantification of the extent of surface modification. Under this consideration, the data shown for reflectivity *vs.* ion fluence shows sharp reduction in optical reflectivity that saturates to ~1% at higher fluences. This would suggest that growth rate of surface features are also saturating, similar to how the growth rate of W fuzz is known to saturate at relatively high fluences^32^. However, dedicated studies involving detailed cross-sectional analysis and height measurements would be needed to fully demonstrate this correlation.

### X-ray Photoelectron Spectroscopy

**Figure 6:**
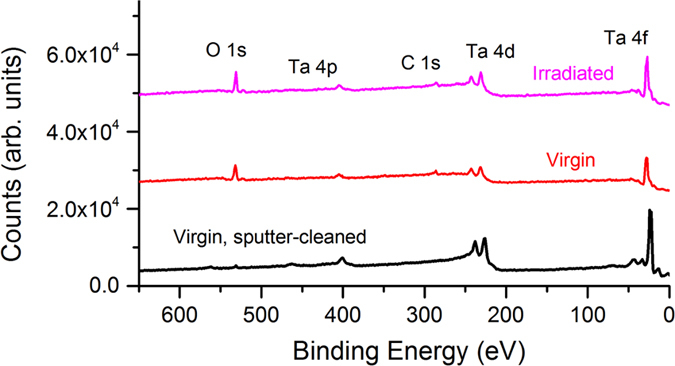
XPS survey spectra for a Ta sample irradiated at 823 K to a fluence of 4.3 × 10^24^ ions m^−2^, referenced against a virgin, mirror-polished Ta samples and a virgin, sputter-cleaned sample.

*Ex situ* XPS has previously been reported by us^19^,^20^ to be a valuable technique not only for determining surface chemistry and oxidation states of samples, but also for indirectly measuring surface roughness post-irradiation. Since a roughened surface has a larger effective surface area, and larger surface area provides increased number of sites available for oxidation, one would expect a very rough surface to oxidize more efficiently when exposed to air. Accordingly, high-resolution XPS analysis of the Ta 4f peak quantitatively reveals how oxidized the Ta surface is and qualitatively gives some information about relative surface roughness. Figure 6 shows an XPS survey spectrum for an irradiated Ta sample (823 K, 4.3 × 10^24^ ions m^−2^) compared against similar spectra for a virgin Ta sample, both before and after a sputter cleaning process. The sputter cleaning process, consisting of 1 keV Ar^+^ at a flux of 10^21^ ions m^−2^ s^−1^ to a fluence of 1.8 × 10^24^ ions m^−2^, was performed *in situ* to remove the native oxide layer and resolve positions of the pure Ta XPS peaks. TRIM^27^, along with some additional calculation, was used to determine the total oxide thickness removed from this sputtering process. Initial TRIM simulations of 1 keV Ar^+^ in stoichiometric Ta_2_O_5_ (with reported density of 8.2 g/cc) shows sputtering yields of 0.37 and 2.70 for Ta and O, respectively. Since O sputters preferentially over Ta, one would expect the steady-state surface stoichiometry to differ form the bulk stoichiometry; this new surface stoichiometry would, in turn, alter the sputtering yield. Using the initial sputter yields obtained from TRIM, and following the procedures outlined in ref. 34, we obtain a steady-state surface stoichiometry of Ta_1.16_O. Inputting this new stoichiometry into TRIM (with calculated density), we obtain new sputtering yields of 0.71 and 1.55 for Ta and O, respectively. It should be noted that the ratio of Ta-to-O sputtering yield closely matches the ratio of Ta-to-O atoms (2-to-5) in the bulk. Therefore, these sputtering yields provide a sufficient approximation of the steady-state sputtering rates for Ta_2_O_5_. Additionally, with the sputtering yields and Ar^+^ ion flux and fluence mentioned above, we calculated that this sputter-cleaning process would have removed ~57 μm of oxide layer. Since this value is well above the anticipated native oxide layer thickness, any remaining oxygen in the Ta matrix is likely a result of some mixing due to Ar^+^ ion irradiation.

**Figure 7:**
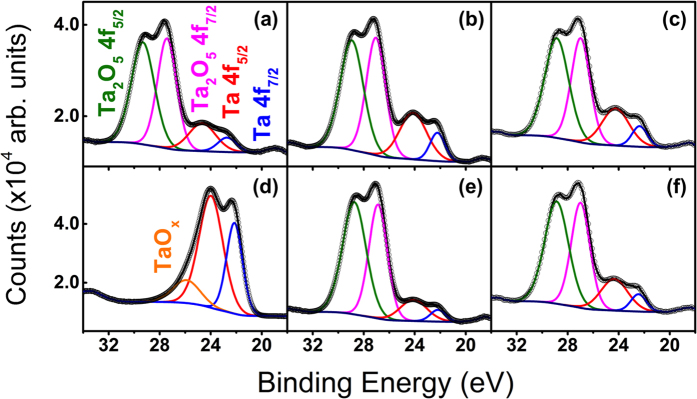
XPS region spectra of the Ta 4f doublet peak at different conditions: (**a**) virgin, before sputter-cleaning; (**b**) 823 K, 4.3 × 10^24^ ions m^−2^; (**c**) 1223 K, 4.3 × 10^24^ ions m^−2^; (**d**) virgin, after sputter-cleaning; (**e**) 823 K, 1.7 × 10^25^ ions m^−2^; (**f**) 1223 K, 1.7 × 10^25^ ions m^−2^.

While survey XPS spectra were taken for several irradiated samples, there is no discernible difference between individual irradiated Ta samples in these low-resolution XPS scans. Aside from the Ta XPS peaks, only C and O peaks were found in any of the XPS spectra; this suggests that there was no additional contamination introduced to the Ta surface during sample preparation or experimentation. In the virgin sputter-cleaned spectrum, only pure Ta peaks are observed with a very small O peak remaining. Once again, this O peak is likely a result of mixing from the sputter cleaning process. Figure 7 shows the high-resolution XPS region spectra of the Ta 4f doublet peak. Each individual spectrum was deconvoluted into four individual mixed Gaussian-Lorentzian peaks (70% Gaussian, 30% Lorentzian) and fit with commercial CasaXPS peak fitting software^35^. Figure 7(a,d) show the spectra from virgin, mirror-polished Ta samples before and after the Ar^+^ sputter-cleaning process, respectively. Here, it is clear that the sputter-cleaning process causes almost complete removal of oxide, evidenced by the pronunciation of pure Ta XPS peaks and the disappearance of Ta_2_O_5_ XPS peaks. It should be noted that there is still some residual peak in the sputter-cleaned spectrum; this peak is attributed to sub-stoichiometric oxides that result from ion beam mixing during the sputtering process. Figure 7(b,c,e,f) show similar region spectra for Ta samples irradiated at: (b) 823 K, 4.3 × 10^24^ ions m^−2^; (c) 1223 K, 4.3 × 10^24^ ions m^−2^; (e) 823 K, 1.7 × 10^25^ ions m^−2^; and (f) 1223 K, 1.7 × 10^25^ ions m^−2^. From these spectra, it is evident that the majority of near-surface Ta is oxidized in the Ta_2_O_5_ state, the most stable and abundant Ta-oxide state. It should be noted that between individual samples, the ratio of oxidized-to-pure peak intensities varies somewhat. Table 1 shows relevant parameters (binding energy and full-width half-maximum (FWHM)) of each individual peak and Table 2 shows the calculated at.% oxidation (ratio of integrated counts from oxide vs. pure peaks) for each sample to quantitatively demonstrate changes in oxidation between samples. The calculations clearly indicate a significant enhancement in oxidation level of Ta samples with increasing He^+^ ion fluence at all temperatures. Since XPS is performed *ex situ*, a more modified surface will have a larger number of potential sites available for oxidation and will oxidize more quickly. Therefore, the increase in oxidation can be used an indirect and qualitative metric to demonstrate increased surface roughness with increasing ion fluence for irradiated samples. These observations corroborate trends observed in both the SEM and optical reflectivity results.

### X-ray Diffraction

**Figure 8:**
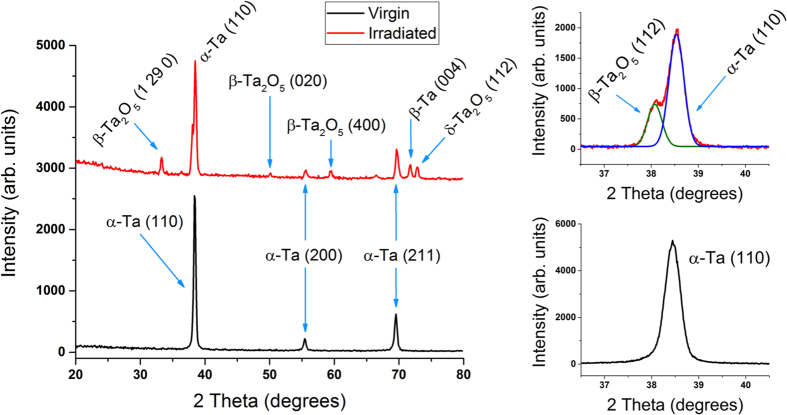
Left: XRD survey spectra of a Ta sample irradiated at 1223 K to a fluence of 3.5 × 10^25^ ions m^−2^, referenced against a virgin, mirror-polished sample. Right: XRD region spectra of the (110) peak for the same samples.

Figure 8 shows survey and (110) region X-ray diffraction (XRD) patterns from virgin and irradiated (1223 K, 3.5 × 10^25^ ions m^−2^) Ta samples. The virgin XRD survey pattern shows only BCC α-Ta XRD peaks (common phase for Ta foil and powder): (110), (200), and (211) corresponding to 2θ values of 38.4, 55.4, and 69.5°, respectively. Note that our virgin Ta samples are polycrystalline (as shown by EBSD) and measured XRD patterns should be expectedly similar to those for Ta powders. On the other hand, irradiated Ta samples show additional Ta_2_O_5_ XRD peaks in addition to pre-existing pure Ta XRD peaks. The majority of Ta_2_O_5_ is in orthorhombic phase, commonly known as β-Ta_2_O_5_. Observed β-Ta_2_O_5_ XRD planes are (1 29 0), (0 2 0), and (4 0 0) corresponding to 2θ values of 33.3, 50.1, and 59.3°, respectively^36^,^37^,^38^. We also notice evidence of hexagonal metastable δ-Ta_2_O_5_ phase with a (1 1 2) XRD plane corresponding to a 2θ value of 72.8° 39. In addition, we also see evidence of tetragonal β-Ta (004) (a metastable phase) at a 2θ value of 71.7° 40. While both irradiated and virgin samples are heavily oxidized (as demonstrated by XPS analysis) the vertical protrusion of surface features on the irradiated sample combined with low angle-of-incidence of the X-ray source ensures a significant XRD signal from the Ta tendril layer. This analysis shows that surface structure evolution involves the formation of new Ta crystal phases. From the high-resolution region patterns, each (110) peak was fitted with a Gaussian peak shape and analyzed (following procedures outlined in refs 41, 42, 43) to determine surface strains. Each Gaussian peak was fit with OriginPro software^44^ and the corresponding strains were determined using the relation 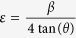 where β is the integral breadth of the XRD peak (β = A/h, where A and h are the peak area and height, respectively) and θ is the corresponding Bragg angle. Using the above analysis, the calculated strain values for (110) α-Ta phase virgin and irradiated Ta samples are 0.0052 and 0.0050, respectively. This indicates that, within experimental uncertainty, there is no significant strain due to ion irradiation in extreme conditions at elevated temperatures.

## Conclusions

In the search for better plasma-facing materials for future nuclear fusion reactors, an important parameter to consider is the material’s response to high-flux, low-energy He^+^ ion irradiation. While W is currently considered the primary candidate material for PFCs in fusion reactors, it has been shown that W undergoes significant surface morphology evolutions when irradiated with low-energy, high-flux He^+^ ion irradiation under certain conditions. This work proposes the use of Ta as an alternative plasma-facing material to W. Mirror-polished Ta samples were irradiated with 100 eV He^+^ ions at a constant flux of 1.2 × 10^21^ ions m^−2^ s^−1^ to fluences in the range 4.3 × 10^24^ – 1.7 × 10^25^ ions m^−2^ at constant sample temperatures in the range 823–1223 K. Resulting surface structures were characterized *ex situ* by a combination of SEM, optical reflectivity, XPS, and XRD. SEM studies reveal that He^+^ ion irradiation, at the highest sample temperature (1223 K), induces nano-scale tendril-like structures similar to the early-stage formation of W “fuzz”. Optical reflectivity measurements show significant enhancement in surface roughness with increasing ion fluence; this agrees well with surface structures observed during SEM studies. XPS and XRD studies confirm the formation of Ta_2_O_5_ phase formation after He^+^ ion irradiation at elevated temperature. While the bulk thermal conductivity and melting point of Ta are both lower than W, this works demonstrates that Ta has a higher fluence threshold for nanostructure formation; this may be a redeeming property for Ta as a plasma-facing material since the reduced appearance of high aspect ratio surface structuring may suggest that the thermal conductivity of Ta would degrade less quickly than W over the lifetime of a fusion reactor. Future work will explore the effect of deuterium (D) irradiation on Ta and Ta surface structures through sequential and simultaneous D^+^ and He^+^ ion irradiation experiments to further assess the relevance of Ta to future fusion devices.

## Methods

All experiments were performed at the UHFI (ultra-high flux irradiation) and IMPACT (interaction of materials with particles and components testing) surface characterization laboratories at CMUXE (center for materials under extreme environment), Purdue University. More details on the UHFI and IMPACT laboratories can be found in refs 45 and 46, respectively. ~10 × 10 mm Ta samples were cut from a 0.5 mm-thick foil (99.95% purity, annealed and cold rolled) and mechanically polished to a mirror-like finish. For an initial characterization of the virgin, mirror-polished samples, atomic force microscopy (AFM) and electron backscatter diffraction (EBSD) were used to determine the material’s initial surface roughness and average grain size, respectively. AFM was performed with a Bruker Innova model AFM in tapping mode, while the EBSD was performed with an FEI XL40 model field emission scanning electron microscope (FE-SEM).

To determine what types of surface structures Ta may develop in a future fusion reactor, all samples were irradiated at normal incidence in an ultra-high vacuum chamber (10^−7^ Torr base pressure) with 100 eV He^+^ while being simultaneously heated by a resistive heater. He^+^ ions for irradiation were produced by a gridless End-Hall type ion source, the EH-400LE, manufactured by Kauffmann & Robinson, Inc. The flux of this broad beam ion source was held constant at 1.2 × 10^21^ ions m^−2^ s^−1^. To compensate for any additional sample heating from the ion source, the resistive heater was equipped with a thermocouple-based temperature feedback loop and PID control such that the sample temperature during each irradiation was constant. To determine both the temperature-dependent and fluence-dependent behavior of nanostructure formation, Ta samples were irradiated at several temperatures in the range 823–1223 K and at several fluences in the range 4.3 × 10^24^ ions m^−2^–1.7 × 10^25^ ions m^2^. Due to interesting surface structures observed at 1223 K irradiations, one additional sample at this temperature was irradiated to a total fluence of 3.5 × 10^25^ ions m^−2^. The resulting surface morphologies of irradiated samples were characterized primarily through field emission scanning electron microscopy (FE-SEM) using the Hitachi S-4800 FE-SEM. Although AFM can typically resolve finer surface features, irradiated samples exhibited large surface roughness, effectively making AFM measurements expectedly difficult. To compensate for loss of depth information that would have been provided by AFM, optical reflectivity was employed to provide some quantitative metric for surface structure evolution. A combination of halogen and deuterium light were used to produce a spectrum of photons in the range 200–1100 nm; reflected photons were collected with Maya 2000 Pro Spectrometers.

To monitor changes in surface chemistry and oxidation states of irradiated Ta samples, X-ray photoelectron spectroscopy (XPS) was performed on several irradiated samples as well as a virgin Ta sample both before and after a sputter cleaning process to remove any native oxide. The sputter cleaning process consisted of irradiating the virgin Ta sample *in situ* with 1 keV Ar^+^ at a flux of 10^21^ ions m^−2^ s^−1^ to a total fluence of 1.8 × 10^24^ ions m^−2^. X-rays for XPS were generated with an Mg-Kα source, and photoelectrons were detected with an Omicron Argus hemispherical analyzer with a round aperture of 6.3 mm; no sample charging was observed. All XPS spectra were analyzed using commercial CasaXPS software^35^. To further characterize near-surface effects of high-flux He^+^ ion irradiation during simultaneous sample annealing, X-ray diffraction (XRD) 2θ scans were produced for virgin and irradiated Ta samples. XRD was performed with a PANalytical X’Pert X-ray diffractometer; Cu-Kα X-rays for XRD bombarded samples at a low angle of incidence (Ω = 5°) such that the XRD signal had a strong contribution from the near-surface of both virgin and irradiated Ta samples. High-resolution scans of high-intensity peaks were then fitted and analyzed with commercial OriginPro software^44^ to realize any changes in surface stress between virgin and irradiated samples.

## Additional Information

**How to cite this article**: Novakowski, T. J. *et al*. Effect of high-flux, low-energy He^+^ ion irradiation on Ta as a plasma-facing material. *Sci. Rep.* **6**, 39746; doi: 10.1038/srep39746 (2016).

**Publisher's note:** Springer Nature remains neutral with regard to jurisdictional claims in published maps and institutional affiliations.

## Figures and Tables

**Table 1:** Binding energies and full-width half-maxima (FWHM) for XPS Ta 4f region peaks shown in Fig. 7.

Sample	Ta 4f_5/2_		Ta 4f_7/2_		Ta2O5 4f_5/2_		Ta2O5 4f_7/2_	
	BE (eV)	FWHM (eV)	BE (eV)	FWHM (eV)	BE (eV)	FWHM (eV)	BE (eV)	FWHM (eV)
Virgin, sputter-cleaned	24.01	2.21	22.15	1.48	26.71	2.5	25.71	1.67
Virgin	24.67	2.5	22.72	1.71	29.31	2.14	27.43	1.77
823 K, 4.3e24 ions m^−2^	24.12	2.5	22.22	1.37	28.94	2.14	27.05	1.77
1023 K, 4.3e24 ions m^−2^	24.14	2.5	22.24	1.38	28.8	2.2	26.94	1.7
1223 K, 4.3e24 ions m^−2^	24.25	2.5	22.36	1.4	28.86	2.21	26.99	1.75
823 K, 1.7e25 ions m^−2^	24.09	2.47	22.19	1.38	28.74	2.18	26.88	1.7
1023 K, 1.7e25 ions m^−2^	24.08	2.43	22.19	1.34	28.85	2.14	26.98	1.71
1223 K, 1.7e25 ions m^−2^	24.34	2.5	22.45	1.41	28.87	2.21	27	1.73

**Table 2:** Calculated pure vs. oxidized concentrations for the XPS region spectra of irradiated samples shown in Fig. 7.

Sample	Total Atomic Concentration (%)	
	Ta	Ta_2_O_5_
823 K, 4.3e24 ions m^−2^	26	74
1023 K, 4.3e24 ions m^−2^	19	81
1223 K, 4.3e24 ions m^−2^	23	77
823 K, 1.7e25 ions m^−2^	14	86
1023 K, 1.7e25 ions m^−2^	14	86
1223 K, 1.7e25 ions m^−2^	20	80
